# Cost-effectiveness of various referral pathways to identify advanced fibrosis among type 2 diabetes mellitus patients with metabolic dysfunction-associated steatotic liver disease in primary care setting in Malaysia

**DOI:** 10.1371/journal.pone.0350263

**Published:** 2026-05-28

**Authors:** Wei Yoon Poh, Amirah Azzeri, Shamsul Mohd Zain, Rosmawati Mohamed, Kim Sui Wan, Xe Hui Lee, Maznah Dahlui, Fatiha Hana Shabaruddin

**Affiliations:** 1 Department of Clinical Pharmacy and Pharmacy Practice, Faculty of Pharmacy, Universiti Malaya, Kuala Lumpur, Malaysia; 2 Department of Pharmacy, Hospital Selayang, Ministry of Health Malaysia, Batu Caves, Selangor, Malaysia; 3 Health Economic Research and Casemix Division, Department of Research and Clinical Advancement, Universiti Malaya Medical Centre, Kuala Lumpur, Malaysia; 4 Department of Primary Care, Faculty of Medicine and Health Sciences, Universiti Sains Islam Malaysia, Nilai, Negeri Sembilan, Malaysia; 5 Department of Pharmacology, Faculty of Medicine, Universiti Malaya, Kuala Lumpur, Malaysia; 6 Department of Medicine, Faculty of Medicine, Universiti Kebangsaan Malaysia, Kuala Lumpur, Malaysia; 7 Centre for Burden of Disease Research, Institute for Public Health, Ministry of Health Malaysia, Shah Alam, Selangor, Malaysia; 8 Endocrinology Unit, Department of Medicine, Hospital Pulau Pinang, Penang, Malaysia; Kwame Nkrumah University of Science and Technology, GHANA

## Abstract

**Background and aim:**

Most international guidelines recommend a two-step approach using the Fibrosis-4 index (FIB-4) and vibration-controlled transient elastography (VCTE) to identify advanced fibrosis, a key predictor of all-cause and liver-related mortality in patients with metabolic dysfunction-associated steatotic liver disease (MASLD). However, VCTE is not available in most primary care settings in Malaysia, and there is scarce data on the cost-effectiveness of different approaches. This study evaluated the cost-effectiveness of three referral pathways for identifying advanced fibrosis among type 2 diabetes mellitus(T2DM) patients with MASLD.

**Methods:**

We developed a decision-analytical model from the healthcare provider’s perspective, using 1,000 simulated patients to compare: (i) Current Practice (direct referral based on elevated alanine transaminase), (ii) Clinical Practice Guidelines (CPG) Pathway using FIB-4 single-cutoff 1.3, and (iii) FIB-4 dual-cutoffs (1.3,3.25) followed by a gamma-glutamyl transferase (GGT) test for indeterminate cases (Sequential FIB-4/GGT Pathway). Current practice served as the reference comparator. The primary outcomes were the average cost-effectiveness ratio (ACER) and the incremental cost-effectiveness ratio (ICER). Model parameters were mainly derived from local studies. Direct medical costs were reported in 2024 Malaysian Ringgit (MYR).

**Results:**

Sequential FIB-4/GGT pathway had the lowest ACER at MYR930 per advanced fibrosis case identified, compared to MYR1,299 for current practice and MYR1,581 for the CPG pathway. Sequential FIB-4/GGT pathway was potentially more effective and less costly, demonstrating dominance over current practice with a cost savings of MYR2,911/additional advanced fibrosis case identified. CPG pathway was more effective and more costly than current practice, with an ICER of MYR3,785.

**Conclusions:**

Sequential FIB-4/GGT pathway was cost-effective for identifying advanced fibrosis in T2DM patients with MASLD. This pragmatic approach could reduce tertiary care referrals, lower healthcare resource use and costs compared to current practice. CPG pathway was more effective than current practice, but incurred higher costs and required increased availability of VCTE within clinical practice.

## Introduction

Metabolic dysfunction-associated steatotic liver disease (MASLD) has emerged as the leading cause of chronic liver disease in adults, with a global prevalence estimated at 38% and reaching as high as 65% among individuals with type 2 diabetes mellitus (T2DM) [1,2]. The coexistence of MASLD and T2DM significantly increases the risk of advanced fibrosis, a key predictor of both all-cause and liver-related mortality, compared to the general T2DM population (23.5% vs. 19.5%) [3,4]. These findings underscore the synergistic effect of T2DM on the progression of liver-related complications and highlight the need for systematic risk assessment of advanced fibrosis in this high-risk population. The clinical burden and economic implications of MASLD are expected to be substantial, driven by the increasing prevalence of the disease and the rise in T2DM worldwide [5,6].

The combination use of several non-invasive tests (NITs), including biochemical markers, fibrosis scoring systems [fibrosis-4 (FIB-4) index, Non-Alcoholic Fatty Liver Disease (NAFLD) fibrosis score (NFS)], and imaging methods such as vibration-controlled transient elastography (VCTE) has been designed and well validated in numerous studies for the evaluation of liver fibrosis [7–12]. Among all NITs, liver stiffness measurement (LSM) by VCTE demonstrates high accuracy in assessing fibrosis stages. However, its availability is often limited to tertiary care facilities due to the need for specialized equipment and trained personnel [13,14].

Most international guidelines, including the American Association for the Study of Liver Diseases (AASLD), the European Association for the Study of the Liver (EASL), and the Asian Pacific Association for the Study of the Liver (APASL), recommend prioritising targeted screening in high-risk populations for MASLD-related advanced fibrosis, particularly among patients with T2DM, using a two-step sequential NITs approach (Eslam, Sarin, et al., 2020; Rinella et al., 2023; Tacke et al., 2024). The Malaysian Clinical Practice Guideline (CPG) for the Management of T2DM supports the same strategy in risk-stratifying advanced fibrosis and added a chapter on MASLD management (W. K. Chan et al., 2022; Ministry of Health Malaysia, 2020). However, since VCTE is only available in tertiary centres, implementing a sequential risk stratification approach using a single cutoff FIB-4 strategy in primary care setting presents challenges due to the high number of patients with indeterminate or high risk of advanced fibrosis who would need to be referred to a tertiary care. To address this gap, our team has recently demonstrated that a sequential use of FIB-4 followed by gamma-glutamyl transferase (GGT) may enhance the stratification of advanced fibrosis in MASLD patients with T2DM who have indeterminate FIB-4 results, while potentially reducing unnecessary referrals to tertiary care [15]. An urgent need therefore exists to evaluate cost-effective referral pathways that optimise resource utilisation within the healthcare system to ensure the rational use of healthcare resources.

This study aimed to analyse the cost-effectiveness of various referral pathways for identifying advanced fibrosis within the Malaysian public primary healthcare system. Evaluating the cost-effectiveness of these pathways in real-world clinical practice will provide healthcare providers with valuable insights into the financial implications, clinical impact, and feasibility of implementing strategies for advanced fibrosis among the high-risk T2DM population with MASLD in the local context. These findings can support evidence-based decision-making and facilitate more effective resource allocation within healthcare systems.

## Materials and methods

### Referral pathways for identifying advanced fibrosis using non-invasive strategies

**Fig 1:**
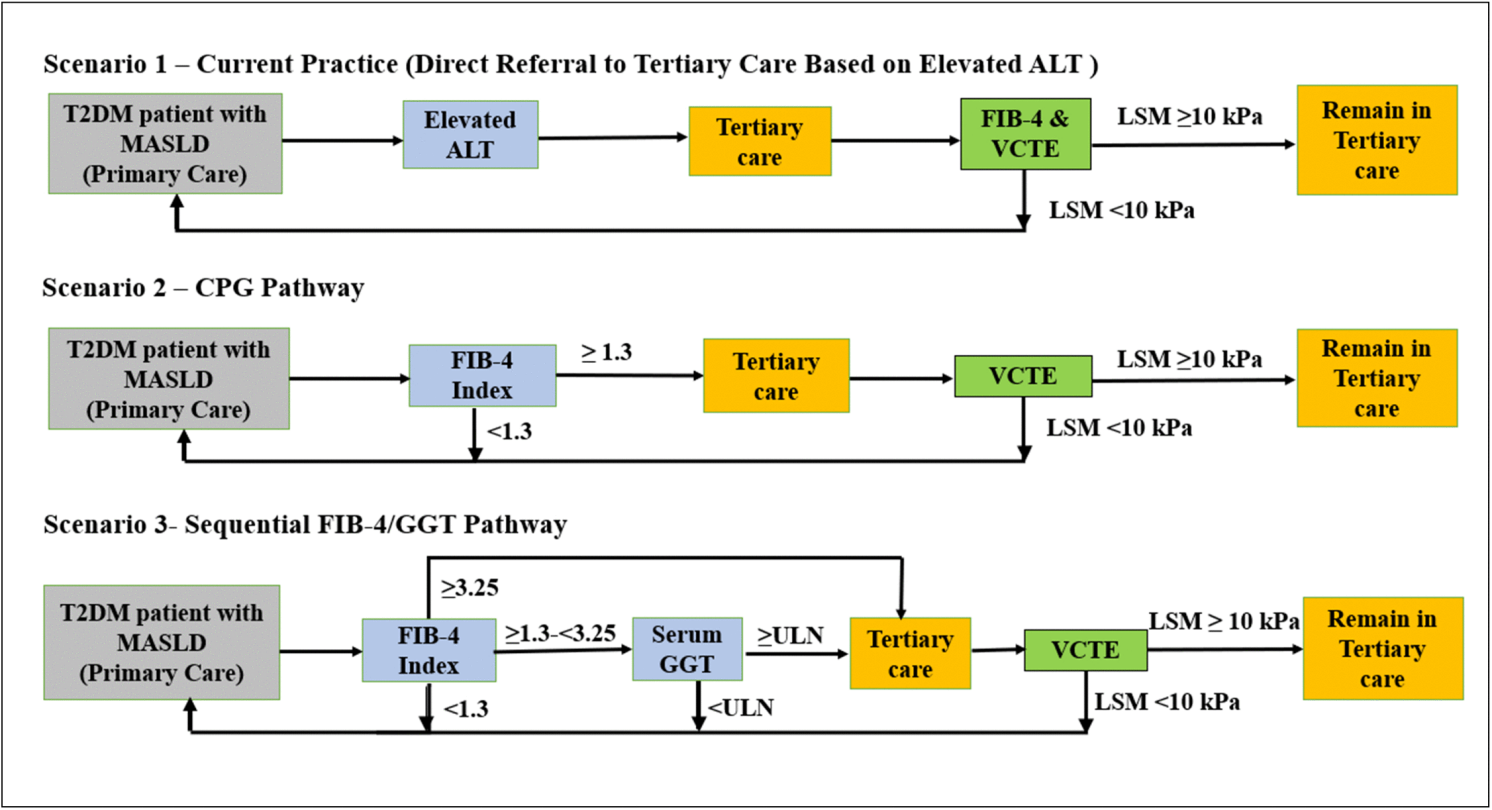
Schematic representation of the three referral pathway scenarios to tertiary care. ALT, alanine aminotransferase; CPG, clinical practice guideline; FIB-4, fibrosis-4 index; GGT, gamma-glutamyl transferase; LSM, liver stiffness measurement; MASLD, metabolic dysfunction-associated steatotic liver disease; T2DM, type 2 diabetes mellitus; ULN, upper limit of normal; VCTE, vibration-controlled transient elastography.

Three referral pathways were analysed (Fig 1). Firstly, the current practice involved referrals to tertiary care from primary care based on elevated serum ALT levels, defined as any level above the upper limit of normal for the individual laboratory cutoff. This served as a reference comparator for this economic evaluation, as informed by interviews with three clinical experts.

The Malaysian national T2DM CPG pathway for risk-stratifying T2DM patients with MASLD using the non-invasive FIB-4 test was assessed as the second referral pathway [16]. The FIB-4 index was calculated using the standard formula: (Age × AST)/ (Platelet Count) × √(ALT), where Age was in years, aspartate aminotransferase (AST) and alanine aminotransferase (ALT) were measured in U/L, and Platelet Count in 10⁹/L [17]. A single FIB-4 cutoff of 1.3 was applied, with scores <1.30 indicating low risk for advanced fibrosis, warranting continued monitoring in primary care, whereas scores ≥1.3 indicated indeterminate or high risk for advanced fibrosis, requiring referral to tertiary care for further assessment. A uniform FIB-4 cutoff of 1.3 was applied across all age groups. Age-adjusted thresholds for individuals aged ≥65 years were not used, as prioritising sensitivity was considered more appropriate to minimise missed cases of advanced fibrosis [18]. For individuals aged <35 years, FIB-4 results should be interpreted with caution and additional non-invasive tests are recommended rather than relying on FIB-4 alone [19].

The third referral pathway involved the sequential use of FIB-4 dual cutoffs, followed by GGT test for indeterminate FIB-4 cases (Sequential FIB-4/GGT Pathway). This strategy was based on our previous evidences demonstrating that sequential use of FIB-4 followed by GGT improves risk stratification for advanced fibrosis in MASLD patients with T2DM who have indeterminate FIB-4 results [15,20]. A FIB-4 score of <1.30 was considered low risk for advanced fibrosis and remained in primary care for monitoring, while a score of ≥3.25 was regarded as high risk and subsequently referred to tertiary care. An indeterminate FIB-4 score between 1.3 and 3.25 was further stratified using the GGT serum test, with an elevated GGT level leading to a referral to tertiary care. Elevated GGT levels were defined as any level above the upper limit of normal for the individual laboratory cutoff.

We assumed all patients tested at risk for advanced fibrosis would be referred to tertiary care for further assessment and that all referred patients would attend their appointments and undergo VCTE to determine their advanced fibrosis status.

### Cost-effectiveness analysis on the referral pathways: Decision analytical model

**Fig 2:**
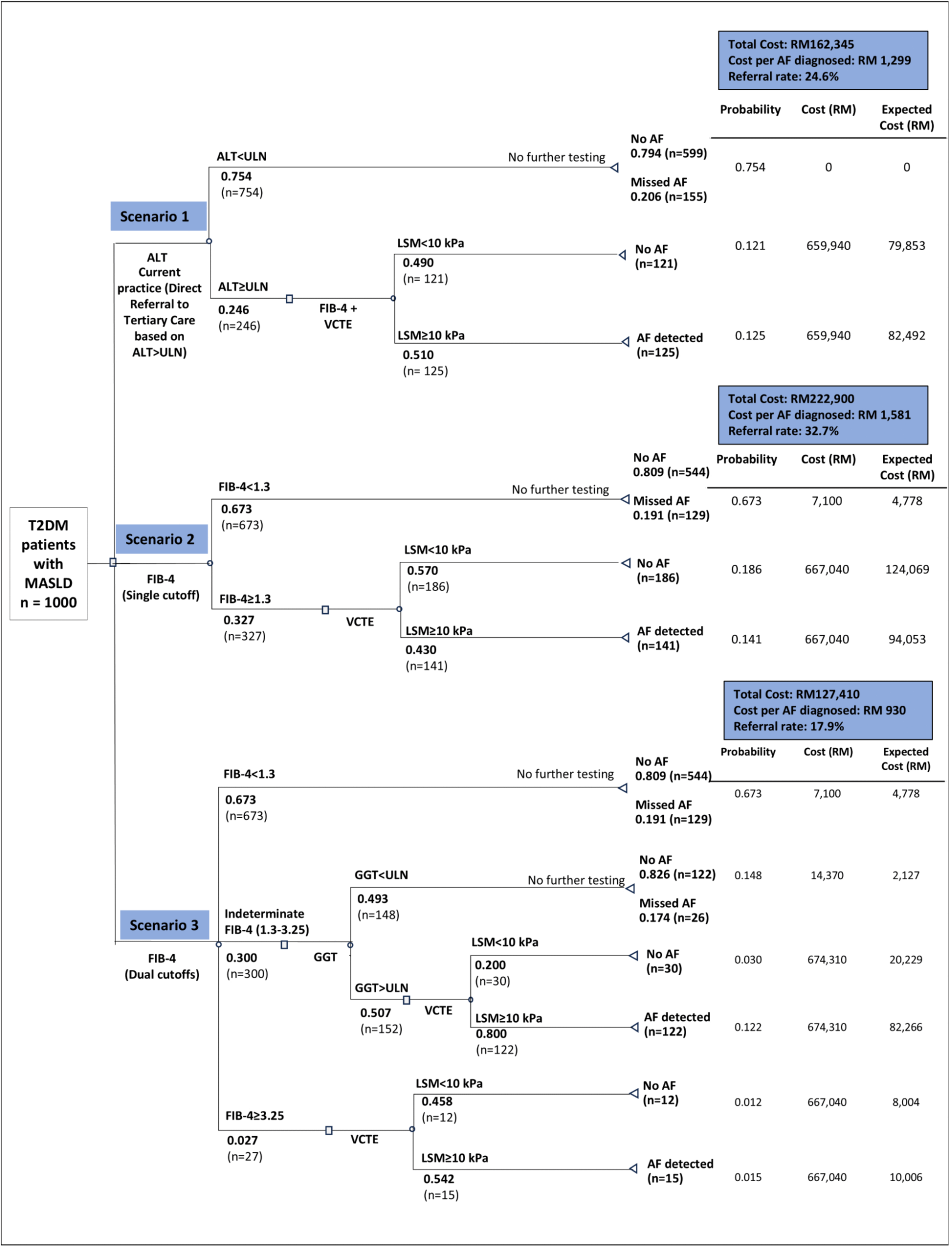
Decision Analytical Model for Various Referral Pathways in Identifying Advanced Fibrosis. AF, advanced fibrosis; ALT, alanine aminotransferase; FIB-4, fibrosis-4 index; GGT, gamma-glutamyl transferase; MASLD, metabolic dysfunction-associated steatotic liver disease; T2DM, type 2 diabetes mellitus; TC, tertiary care; ULN, upper limit of normal; VCTE, vibration-controlled transient elastography.

A decision-analytical model was developed to evaluate these three referral pathways (Fig 2), with a hypothetical cohort of 1,000 T2DM patients with MASLD. T2DM patients were defined by documented T2DM history or the need for diabetic treatment and MASLD was diagnosed based on biochemical markers, histology, or ultrasonography in primary care settings. The healthcare provider perspective was used, and costs were reported in 2024 Malaysian Ringgit (MYR), at the currency exchange rate of USD 1 = MYR 4.4755 (as of December 31, 2024, Central Bank of Malaysia, Bank Negara Malaysia). Discounting was not applied as the time horizon of this analysis was less than 1 year up to the identification of advanced fibrosis, defined as LSM ≥ 10kPa on VCTE. The primary outcomes of this cost-effectiveness analysis were the average cost-effectiveness ratio (ACER) and the incremental cost-effectiveness ratio (ICER) [21]. ACER is calculated as the mean cost per advanced fibrosis case identified, while ICER is the additional cost required per additional advanced fibrosis case identified.

This study is reported based on the Consolidated Health Economic Evaluation Reporting Standards (CHEERS) 2022 Checklist [22] (S1 Appendix). University Malaya Medical Centre Medical Research Ethics Committee (UMMC MREC) approved this study (MREC ID No: 2023628−12609).

### Model input parameters

All input parameters used in the decision-analytical model were collected between 13/09/2024 and 18/03/2025. All input parameters were summarised in S1 Table.

#### Diagnostic performance.

Literature sources from local and Asian countries were prioritised to populate the parameters in our decision-analytical model related to diagnostic performance. i) A nationwide community-based cross-sectional survey on the prevalence of MAFLD in Malaysia [23] (unpublished raw data provided by author, 8 November 2024), ii) a cross-sectional study on 577 T2DM patients at a diabetes clinic in a local university hospital [24] (Unpublished raw data provided by author, 18 March 2025) and iii) a multicentre retrospective analysis of data collected from the Gut and Obesity in Asia Workgroup on the use of NITs to identify advanced fibrosis among biopsy-proven NAFLD patients. Data from the subpopulation with 10% advanced fibrosis, as determined by liver biopsy, were selected to simulate the T2DM population seen in primary care settings [25].

#### Resource utilisation and costs.

Data on referral pathways and resource use within primary and tertiary care settings for identifying advanced fibrosis among T2DM patients with MASLD were collected through interviews with local experts. The cost of each referral pathway was calculated based on all healthcare resources required along each pathway, including direct healthcare costs associated with primary care visits, follow-up reviews, tertiary care visits, and diagnostic investigations, such as laboratory and procedural tests. Cost data were derived from a study on chronic liver disease due to Hepatitis C in Malaysia, based on top-down and bottom-up costing methods, within the public healthcare system [26]. All costs were adjusted to the price of 2024 using an online inflation calculator by the Department of Statistics Malaysia [27].

Costs of managing comorbidities were excluded based on the assumption that patient’s comorbidities, such as T2DM, hypertension, and dyslipidaemia, would be managed clinically regardless of the modelled care pathway. Routine blood investigations such as full blood count (FBC) and liver function test (LFT) for regular monitoring of T2DM patients in primary care were also excluded from the cost analysis. Aspartate aminotransferase (AST) and gamma-glutamyl transferase (GGT) are not part of a standard LFT panel and require specific requests; thus, these costs were included in the pathway cost calculation.

### Sensitivity analysis

One-way deterministic sensitivity analysis was performed, along with probabilistic sensitivity analysis (PSA) using Monte Carlo sampling methods with 1,000 simulations conducted to evaluate the robustness of the results. A cost-effectiveness acceptability curve (CEAC) was generated for each referral pathway to illustrate the probability of cost-effectiveness across a range of willingness-to-pay (WTP) thresholds for identifying one additional case of advanced fibrosis. All analyses were performed using Microsoft Excel® 2019.

## Results

### Cost-effectiveness analysis

CPG Pathway exhibited the highest cost of MYR 222,900, followed by current practice at MYR 162,345, while sequential FIB-4/GGT pathway reported the lowest cost at MYR 127,410 for every 1,000 patients. Current practice had the lowest number of advanced fibrosis cases detected (125 cases), with an ACER of MYR 1299 per advanced fibrosis case identified, while CPG pathway identified the most cases (141 cases) but with the highest ACER at MYR1,581. Sequential FIB-4/GGT pathway demonstrated a high detection yield of 137 cases, and the lowest ACER value of MYR930.

**Fig 3:**
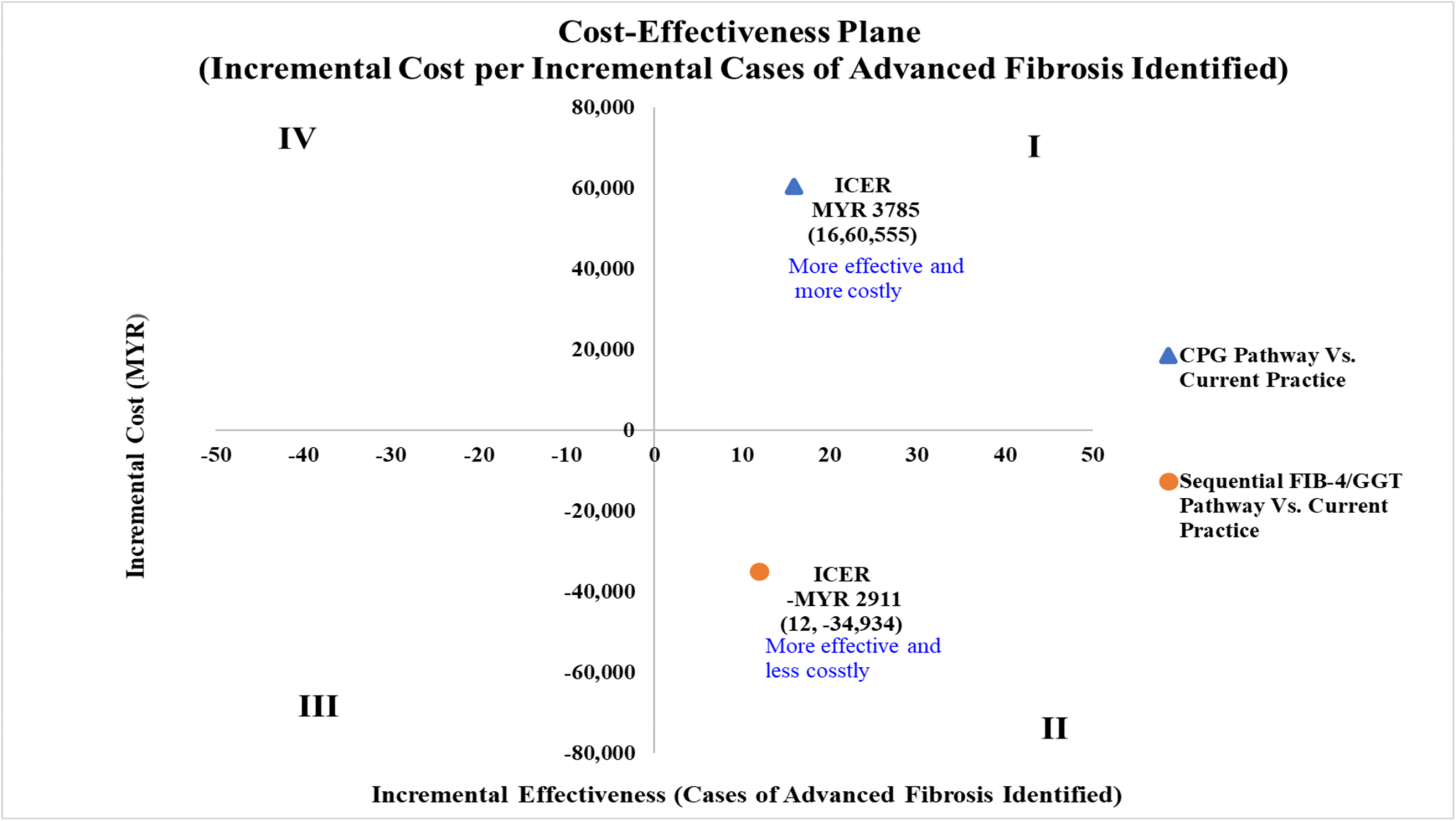
Cost-effectiveness plane for referral pathways in identifying advanced fibrosis in T2DM patients with MASLD (in 1,000 Simulated Patients). All cost values are 2024 Malaysian Ringgit (MYR). ICER, incremental cost-effectiveness ratio; CPG, clinical practice guideline; FIB-4, fibrosis-4 index; GGT, gamma-glutamyl transferase.

The CPG pathway showed an incremental cost of MYR 60,555 and an incremental effectiveness of 16 advanced fibrosis cases identified compared to current practice, resulting in an ICER value of MYR 3,785 per additional advanced fibrosis case identified. In contrast, sequential FIB-4/GGT pathway, demonstrated cost savings of MYR 34,935 and an incremental effectiveness of 12 advanced fibrosis cases identified, indicating more effective, less costly, with a cost saving of MYR 2,911 per additional advanced fibrosis case identified. The results are summarised in Table 1 and the cost-effectiveness plane is illustrated in Fig 3. The CPG pathway compared was more effective yet more costly than current practice. The sequential FIB-4/GGT pathway compared was more effective and less costly than current practice.

**Table 1:** Total and incremental cost-effectiveness results of various referral pathways (in 1,000 Simulated Patients) using base values vs mean values from probabilistic sensitivity analysis.

Intervention	Total			Incremental		Interpretation, (ICER in MYR)
	Costs (MYR)	Advanced Fibrosis Cases Identified (n)	ACER (MYR)	Costs (MYR)	Advanced Fibrosis Cases Identified (n)	
**Base Values**						
Current Practice	162,345	125	1299	–	–	–
CPG Pathway	222,900	141	1581	60,555	16	More effective, more costly, (3,785)
Sequential FIB-4/GGT Pathway	127,410	137	930	−34,935	12	More effective, less costly, (−2,911)
**Probabilistic Sensitivity Analysis (Mean Values)**						
Current Practice	162,621	126	1291	–	–	–
CPG Pathway	223,566	141	1586	60,946	15	More effective more costly, (4,063)
Sequential FIB-4/GGT Pathway	127,781	137	933	−34,840	11	More effective, less costly, (−3,167)

All cost values are 2024 Malaysian Ringgit (MYR).

Negative ICERs are presented with further interpretation of their different meanings (less effective and less costly, more effective and less costly, less effective and more costly).

ACER, average cost-effectiveness ratio; ALT, alanine aminotransferase; CPG, clinical practice guidelines; FIB-4, fibrosis-4 index; GGT, gamma-glutamyl transferase; ICER, incremental cost-effectiveness ratio; ULN, upper limit of normal; VCTE, vibration-controlled transient elastography.

### Impact of various referral pathways for identifying advanced fibrosis in T2DM patients with MASLD

**Fig 4:**
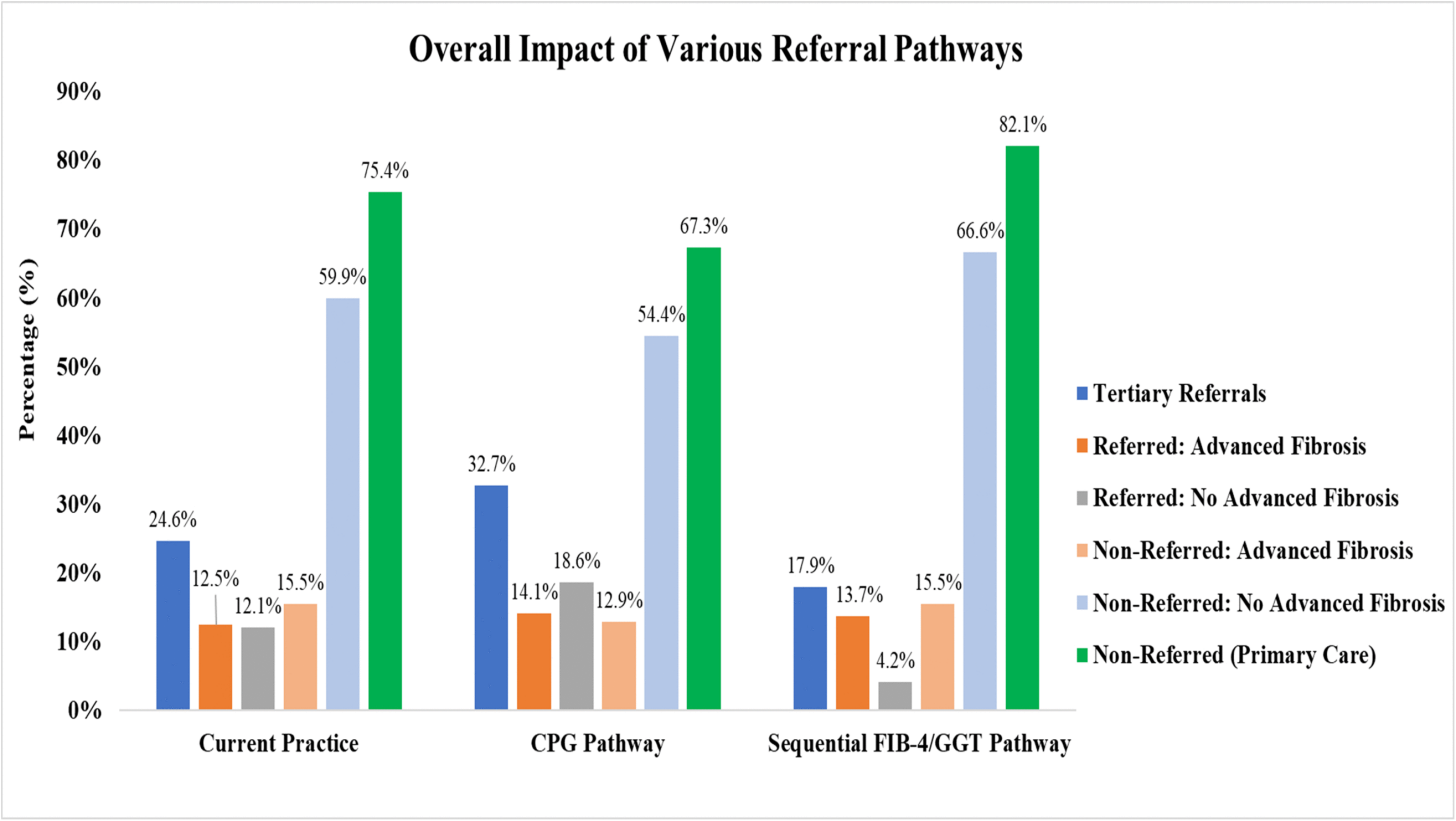
Overall impact of various referral pathways. Referred Case – No Advanced Fibrosis: patients referred to tertiary care but not subsequently identified as having advanced fibrosis (Over-referral). Non-Referred Case – Advanced Fibrosis: patients not referred to tertiary care but who actually have advanced fibrosis [Missed advanced fibrosis]. ALT, alanine transaminase; CPG, clinical practice guideline; FIB-4, Fibrosis-4; GGT, gamma-glutamyl transferase.

**Fig 5:**
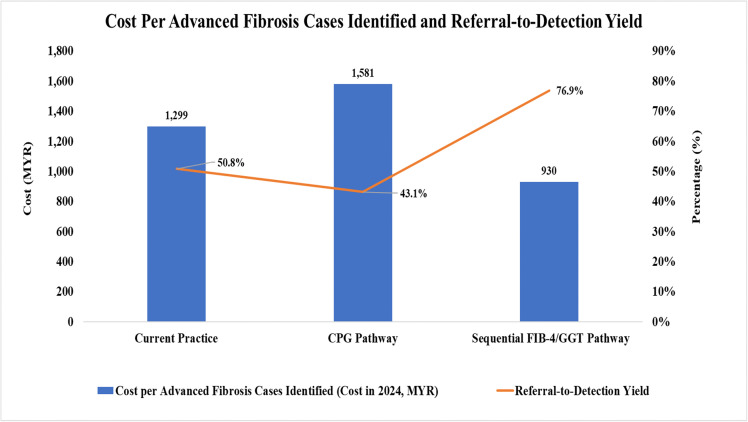
Cost per advanced fibrosis identified and referral-to-detection yield. All cost values are 2024 Malaysian Ringgit (MYR). ALT, alanine transaminase; CPG, clinical practice guideline; FIB-4, Fibrosis-4; GGT, gamma-glutamyl transferase.

Figs 4 and 5 summarise the costs and number of referrals to tertiary care across the three referral pathways. The sequential FIB-4/GGT pathway yielded the lowest referral rate at 17.9%, compared to 24.6% with current practice and 32.7% with the CPG pathway. All three pathways identified a similar proportion of advanced fibrosis cases among referred patients (12.5% to 14.1%). The sequential FIB-4/GGT pathway also demonstrated the lowest over-referral rate at 4.2%, defined as patients referred to tertiary care but not subsequently identified as having advanced fibrosis. In comparison, over-referral rates were 12.1% with current practice and 18.6% with the CPG pathway. The sequential FIB-4/GGT pathway achieved the highest proportion of patients who were not referred to tertiary care and did not have advanced fibrosis (66.6%). All referral pathways showed comparable rates of potentially missed advanced fibrosis cases, represented as patients not referred to tertiary care but identified as having advanced fibrosis, ranging from 12.9% to 15.5%. Notably, the referral-to-detection yield rate was highest with the sequential FIB-4/GGT pathway at 76.9%, markedly higher than current practice (50.8%) and the CPG pathway (43.1%).

### Sensitivity analysis

The tornado diagrams and cost-effectiveness plane presented visual representations of the impact of varying model parameters, ranging from minimum to maximum values in the one-way deterministic sensitivity analysis for both cost and effectiveness (S2 and S3 Tables).

**Fig 6:**
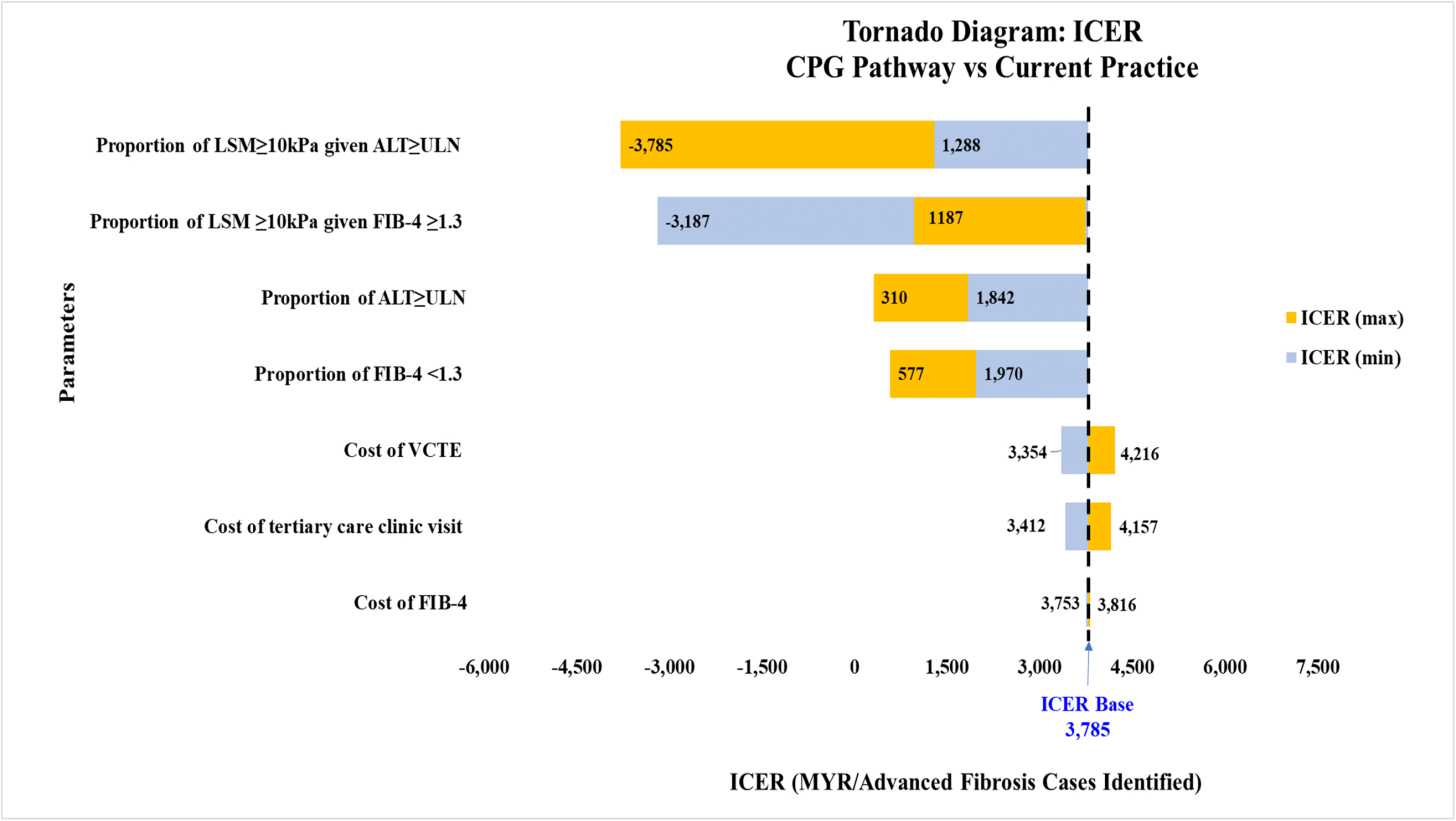
Tornado diagram comparing the CPG pathway with current practice. All cost values are 2024 Malaysian Ringgit (MYR). ALT, alanine transaminase; CPG, clinical practice guidelines; FIB-4, Fibrosis-4; ICER, incremental cost-effectiveness ratio; LSM, liver stiffness measurement; ULN, upper limit normal; VCTE, vibration-controlled transient elastography.

**Fig 7:**
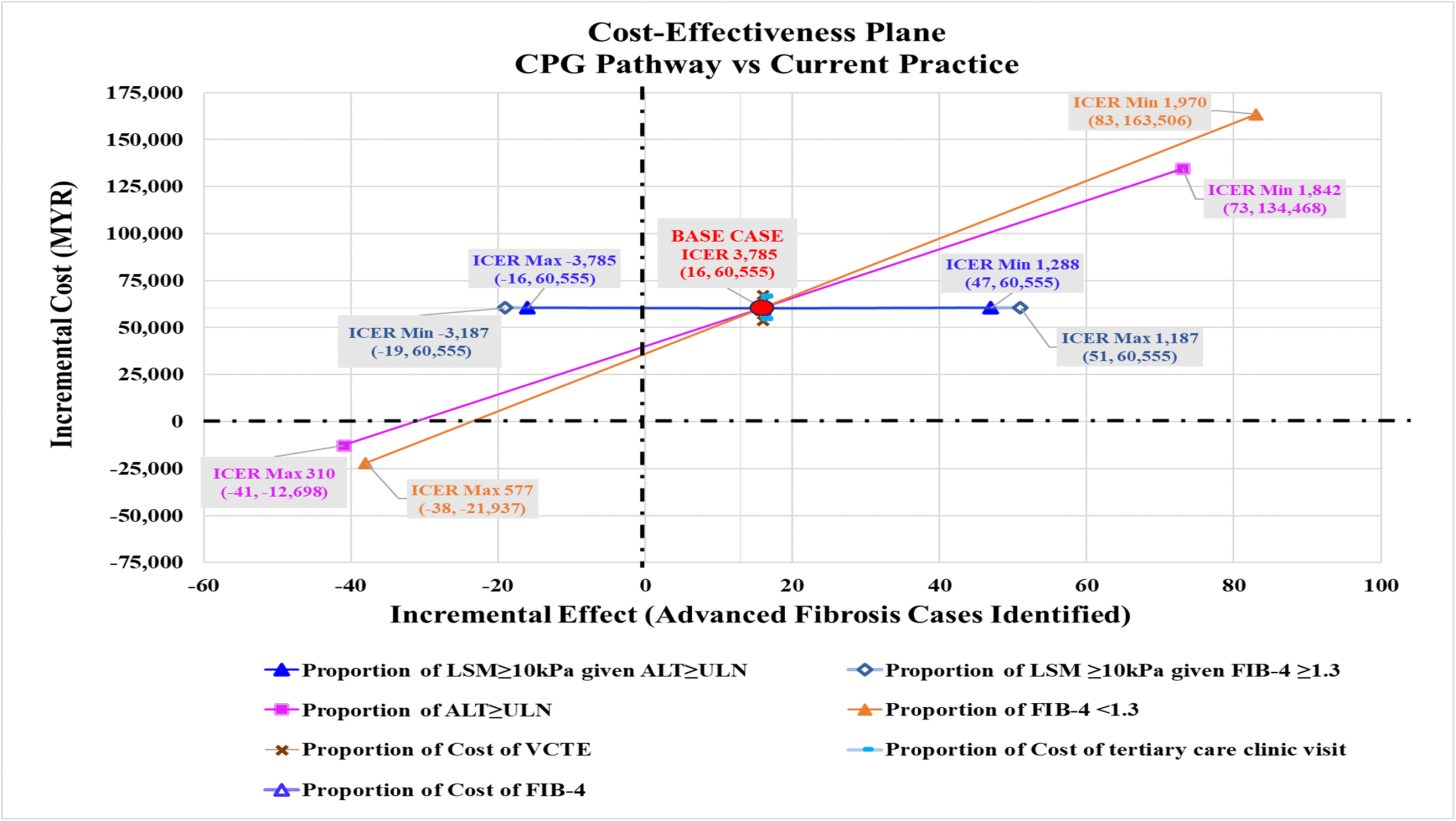
Cost-effectiveness plane comparing the CPG pathway with current practice. Coordinates represent incremental effect and incremental cost values. All cost values are 2024 Malaysian Ringgit (MYR). ALT, alanine transaminase; CPG, clinical practice guidelines; FIB-4, Fibrosis-4; ICER, incremental cost-effectiveness ratio; LSM, liver stiffness measurement; ULN, upper limit normal; VCTE, vibration-controlled transient elastography.

For CPG pathway compared to current practice, the three most influential variables were (i) the proportion of advanced fibrosis given elevated ALT, (ii) the proportion of advanced fibrosis given FIB-4 ≥ 1.3, and (iii) the proportion of elevated ALT (Fig 6). When the proportion of advanced fibrosis given FIB-4 ≥ 1.3 was varied from its minimum value of 0.383 to its maximum value of 0.638, there was a shift in the ICER quadrant from a positive ICER value of MYR1,288 (more effective, same cost) to a negative ICER value of -MYR3,785 (less effective, same cost) (Fig 7). The CPG pathway remains more effective in identifying advanced fibrosis if the proportion of advanced fibrosis, given an elevated ALT is below 0.55 (S1 Fig).

**Fig 8:**
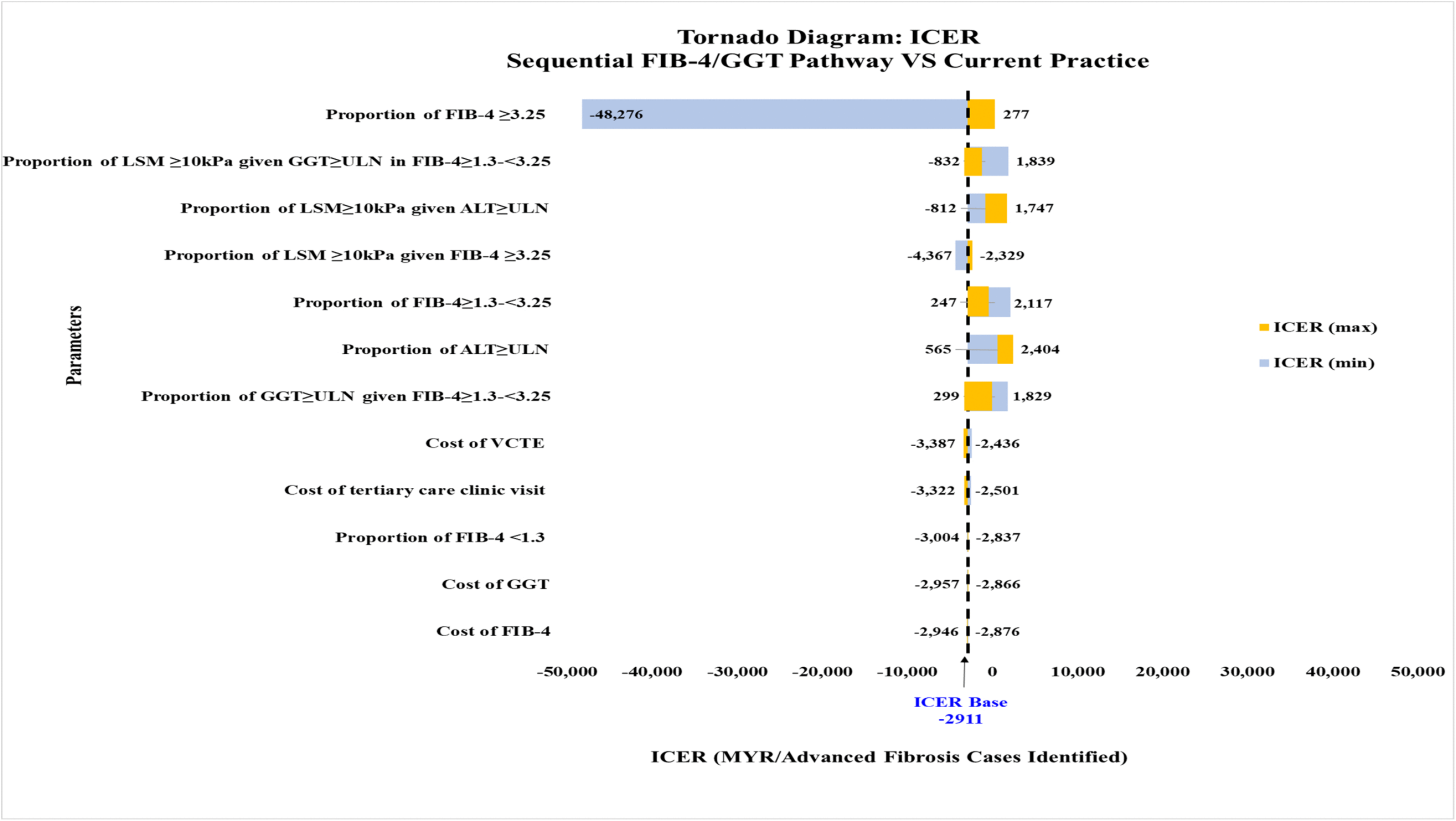
Tornado diagram comparing the sequential FIB-4/GGT pathway with current practice. All cost values are 2024 Malaysian Ringgit (MYR). ALT, alanine transaminase; CPG, clinical practice guidelines; FIB-4, Fibrosis-4; GGT, gamma-glutamyl transferase; ICER, incremental cost-effectiveness ratio; LSM, liver stiffness measurement; ULN, upper limit of normal; VCTE, vibration-controlled transient elastography.

**Fig 9:**
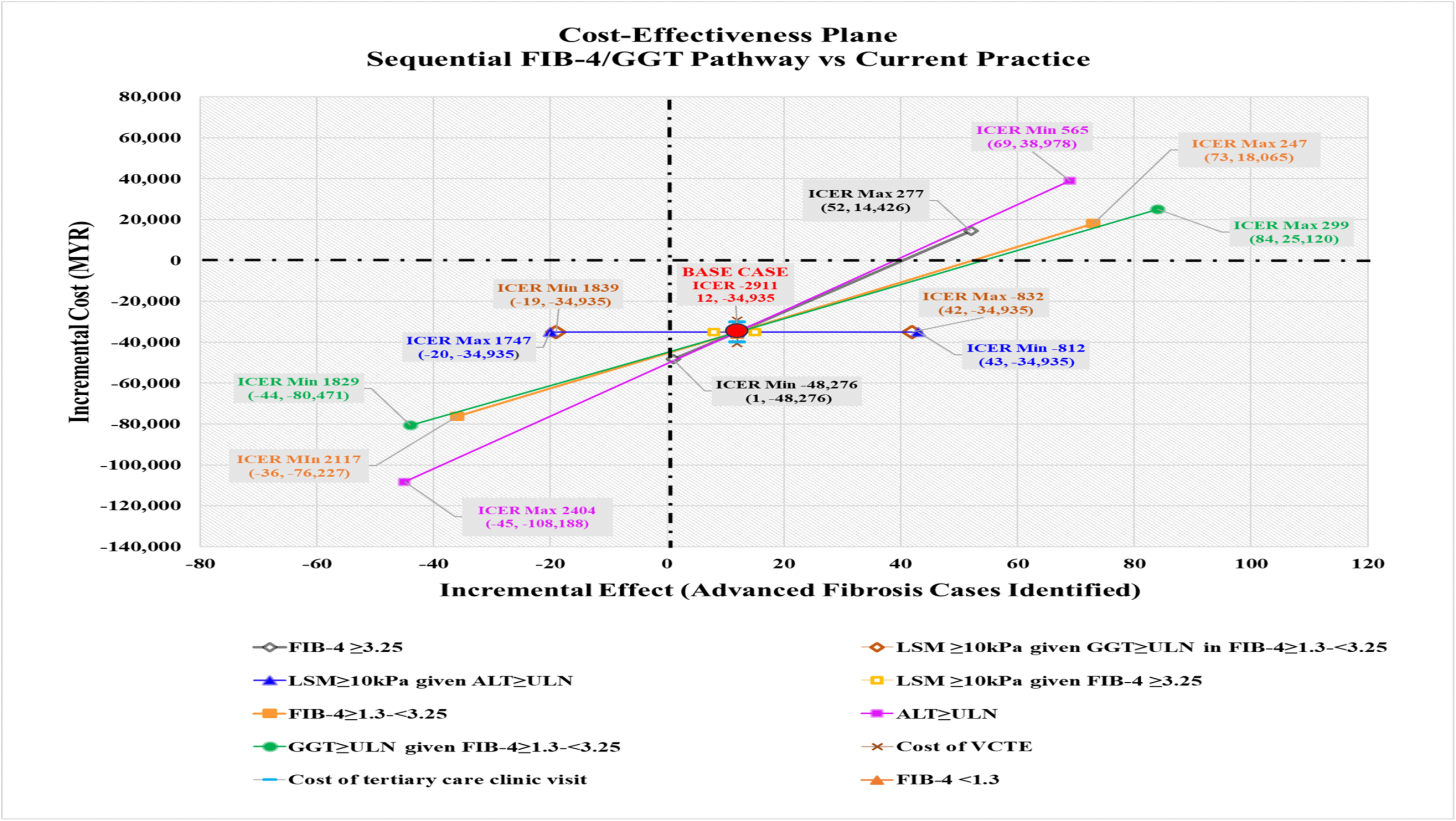
Cost-effectiveness plane comparing the sequential FIB-4/GGT pathway with current practice. Coordinates represent incremental effect and incremental cost values. All cost values are 2024 Malaysian Ringgit (MYR). ALT, alanine transaminase; CPG, clinical practice guidelines; FIB-4, Fibrosis-4; GGT, gamma-glutamyl transferase; ICER, incremental cost-effectiveness ratio; LSM, liver stiffness measurement; ULN, upper limit of normal; VCTE, vibration-controlled transient elastography.

For sequential FIB-4/GGT pathway compared to current practice, the variables influencing the model were (i) the proportion of FIB-4 ≥ 3.25, (ii) the proportion of advanced fibrosis given elevated GGT among those with indeterminate FIB-4, and (iii) the proportion of advanced fibrosis given elevated ALT (Fig 8). When the proportion of FIB-4 ≥ 3.25 was varied from 0.007 (minimum) to 0.101 (maximum), there was a significant shift in the ICER quadrant from a negative ICER value of -MYR 48,276 (less effective, less costly) to a positive ICER value of MYR 277 (more effective, more costly) (Fig 9) due to the incremental number of advanced fibrosis cases identified increasing from 1 to 52 cases. For the FIB-4/GGT pathway to be more effective and less costly than the current practice, the proportion of advanced fibrosis with FIB-4 ≥ 3.25 should be between 0.007 to 0.075 (S2 Fig). Both deterministic sensitivity analyses were minimally affected by cost variables.

**Fig 10:**
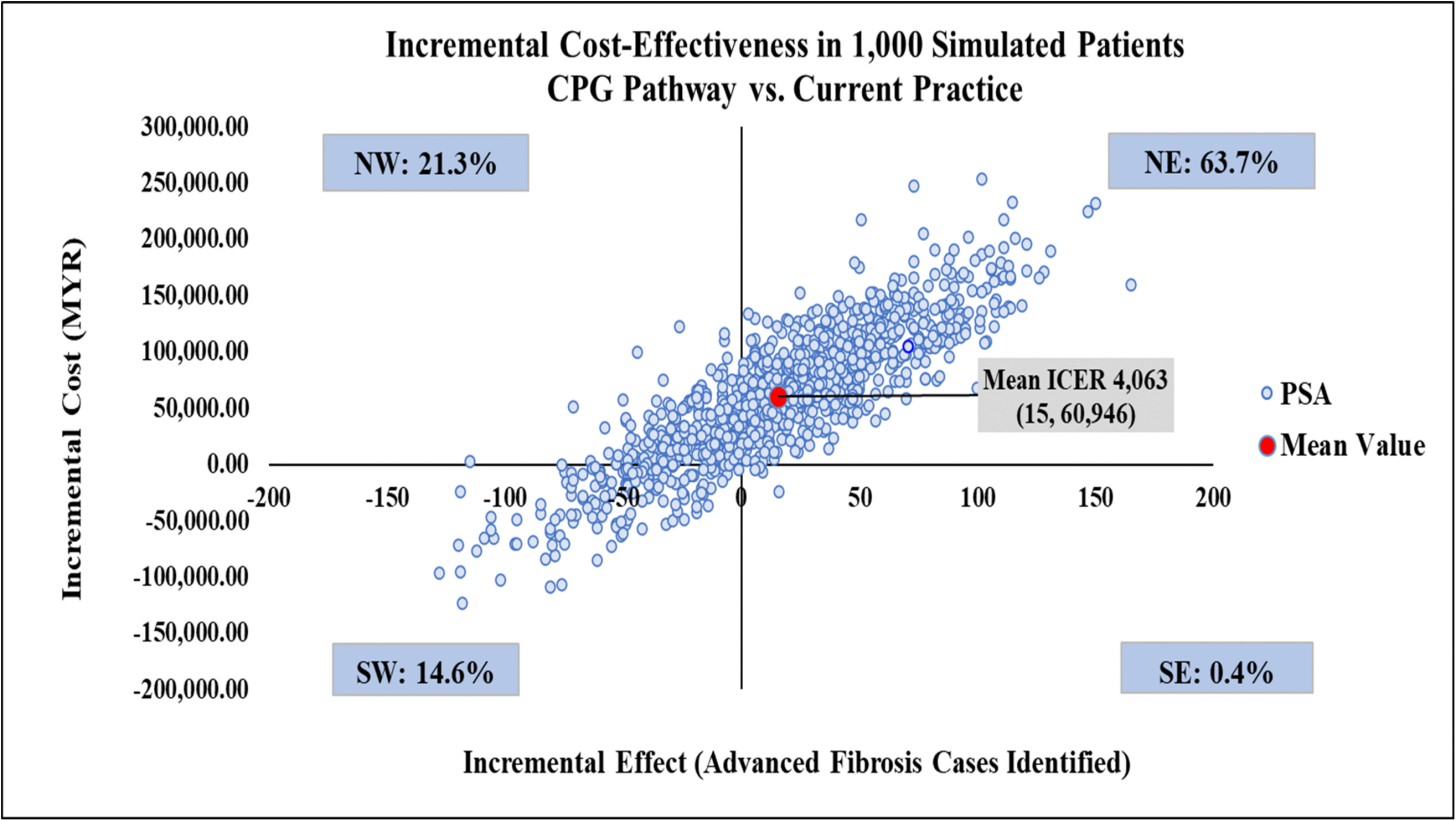
Incremental cost-effectiveness scatter plot comparing the CPG pathway with current practice. CPG, clinical practice guidelines; ICER, incremental cost-effectiveness ratio; NE, north-west; NW, north-west; PSA, probabilistic sensitivity analysis; SE, south-east; SW, south-west.

**Fig 11:**
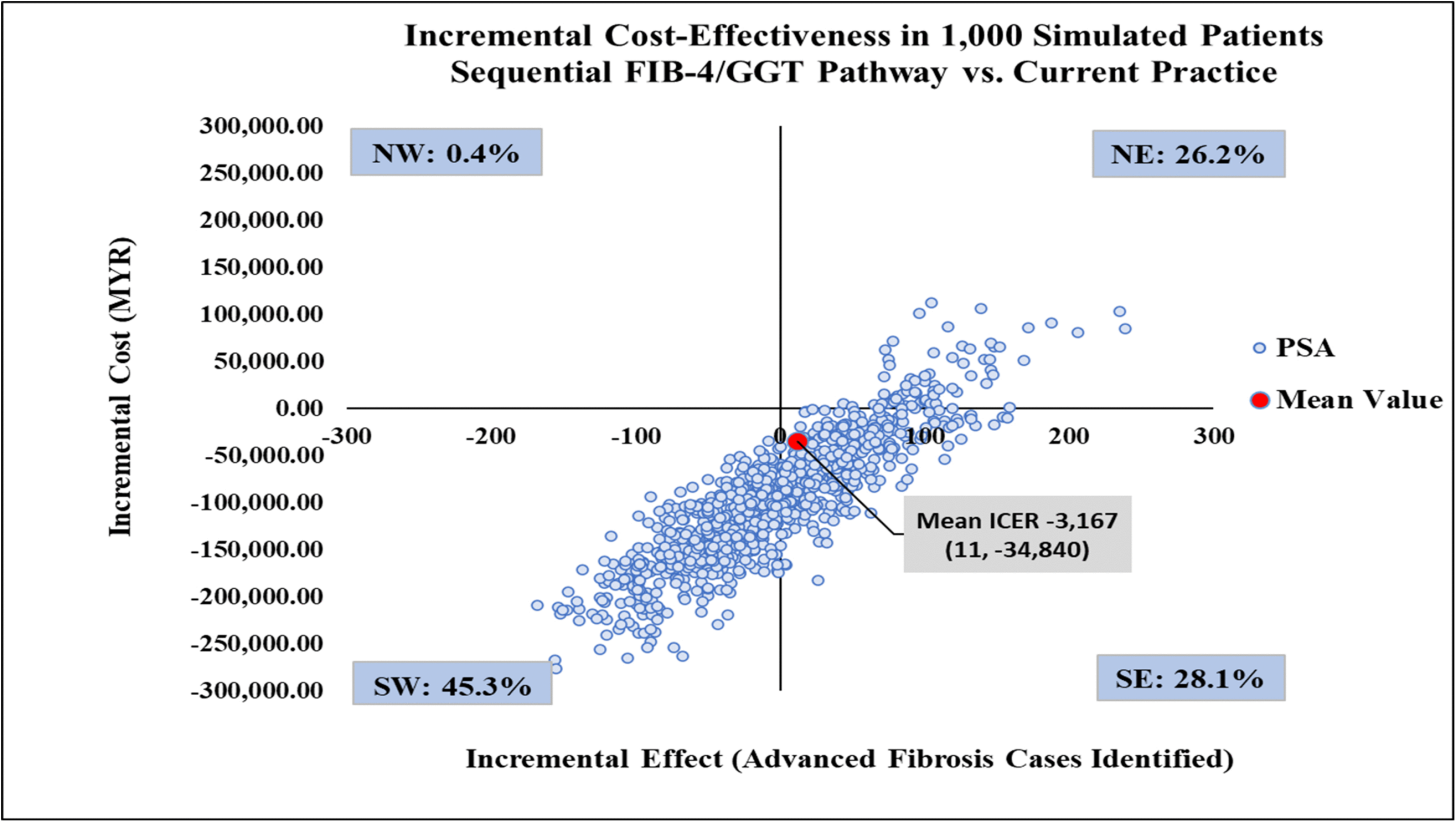
Incremental cost-effectiveness scatter plot comparing the sequential FIB-4/GGT pathway with current practice. CPG, clinical practice guidelines; FIB-4, Fibrosis-4; GGT, gamma-glutamyl transferase; ICER, incremental cost-effectiveness ratio; NE, north-west; NW, north-west; PSA, probabilistic sensitivity analysis; SE, south-east; SW, south-west.

**Fig 12:**
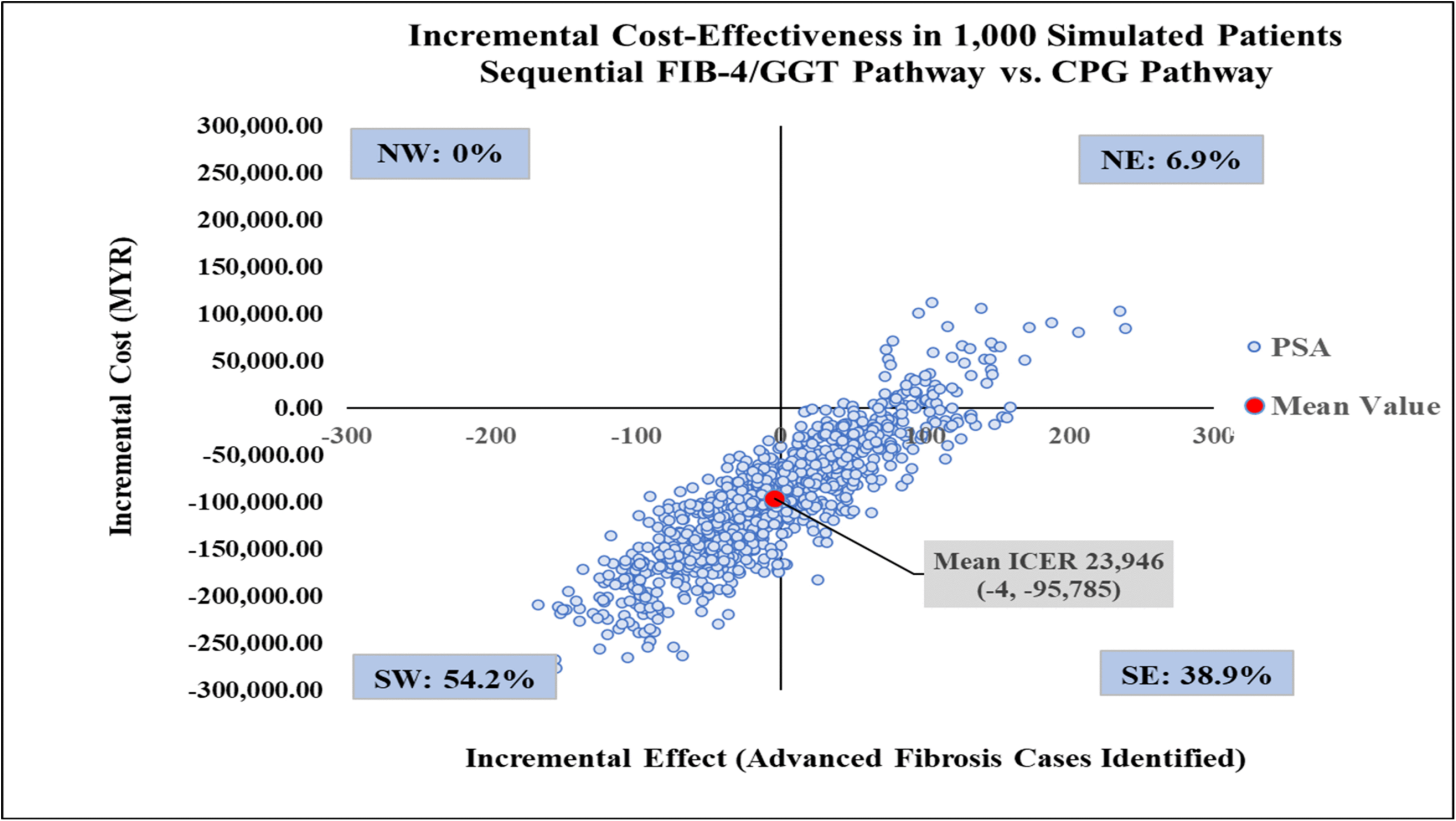
Incremental cost-effectiveness scatter plot comparing the sequential FIB-4/GGT pathway with CPG pathway. CPG, clinical practice guidelines; FIB-4, Fibrosis-4; GGT, gamma-glutamyl transferase; ICER, incremental cost-effectiveness ratio; NE, north-west; NW, north-west; PSA, probabilistic sensitivity analysis; SE, south-east; SW, south-west.

The mean ICER from the probabilistic sensitivity analysis data matched the results from the deterministic base case, reinforcing model robustness (Table 1). The CPG pathway was more effective yet more costly than current practice, with mean ICER of MYR 4,063 per additional advanced fibrosis case identified (Fig 10) and 63.7% of simulated ICERs failing in the northeast quadrant of the CE plane. Meanwhile, the FIB-4/GGT pathway was less effective and less costly than the current practice 45.3% of the time with 28.1% of simulations falling in the southeast quadrant. This suggested that it was potentially more effective and less costly, saving MYR 3,167 per additional advanced fibrosis case identified (Fig 11), making it a favourable option. A further comparison of the FIB-4/GGT pathway with the CPG pathway showed most simulations (54.2%) in the southwest quadrant, indicating it was less effective and less costly, with a mean ICER of MYR 23,946. Approximately 39% of simulations were in the southeast quadrant, indicating a favourable option that is both more effective and less costly (see Fig 12).

**Fig 13:**
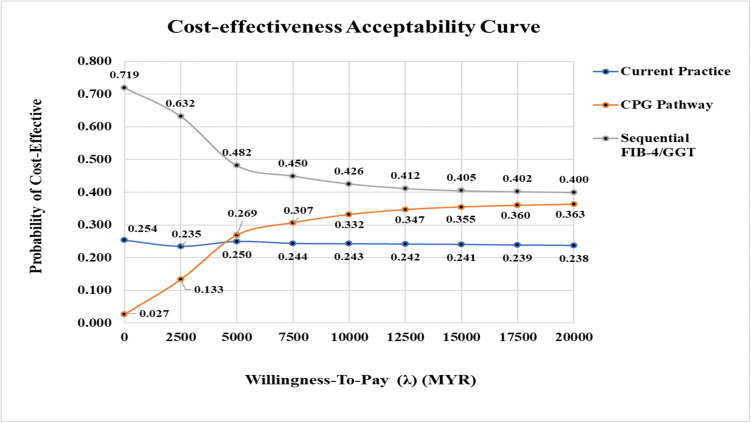
Cost-effectiveness acceptability curve (CEAC) of multiple pathways. CPG, clinical practice guidelines; FIB-4, Fibrosis-4; GGT, gamma-glutamyl transferase; ICER, incremental cost-effectiveness ratio.

The CEAC demonstrated that the sequential FIB-4/GGT pathway had the highest probability of being cost-effective across all WTP thresholds (Fig 13). At a WTP of zero, this pathway showed a 71.9% probability of being cost-effective, compared to 25.4% for current practice and 2.7% for the CPG pathway. A crossover point between the CPG pathway and current practice occurred at approximately MYR 4607, beyond which the CPG pathway became more favourable than current practice. A hypothetical WTP threshold of MYR 5,000 per additional advanced fibrosis case identified was used, the FIB-4/GGT pathway would potentially be the most cost-effective option with a 48.2% likelihood of being cost-effective, compared to 26.9% for the CPG pathway and 25% for current practice.

## Discussion

This study found that the sequential FIB-4/GGT pathway appears to be the most cost-effective strategy compared to current practice and the CPG pathway. The current practice of direct referral to tertiary care based on elevated ALT is a less accurate surrogate marker for severe liver disease, as previous studies have shown that ALT has limited utility in predicting advanced fibrosis [28–30]. A study involving 16,373 patients in primary care in Scotland found that a third of MASLD patients with advanced fibrosis or cirrhosis had low-range ALT levels, which would be missed using the conventional ALT range [31]. This is similar to the findings from a study involving 222 patients in a tertiary university hospital in the United States, which reported that nearly one-third of individuals had non-alcoholic steatohepatitis (NASH) or advanced fibrosis despite normal ALT levels, while more than 50% showed no signs of NASH or advanced fibrosis even with elevated ALT levels [28]. These findings suggest that advanced fibrosis can occur in patients with normal ALT levels, which may lower suspicion for further investigation and delay detection. Therefore, ALT should not be solely relied upon as a referral indicator for advanced fibrosis.

Compared to current practice, the sequential FIB-4/GGT pathway was both more effective and less costly, whereas the CPG pathway was more effective but also more costly. The lower referral rates and comparable number of advanced fibrosis identified through the sequential FIB-4/GGT pathway were the main factors contributing to the cost and outcome differences. Although the CPG pathway detected four more cases of advanced fibrosis than the FIB-4/GGT pathway, over half (57%) of the cases referred through the CPG pathway did not have advanced fibrosis, indicating a high rate of over-referral, as these patients could have been effectively managed in primary care. A UK base case analysis for 1000 NAFLD patients demonstrated that a sequential approach using non-invasive tests to stratify patients with MASLD in routine primary care has reduced unnecessary hospital referrals by as much as 70%, while decreasing total healthcare spending by up to 25% compared to the standard of care that relied on patient history, physical examination, ultrasound and blood tests including LFTs (Srivastava et al., 2019).

The sequential use of FIB-4 followed by VCTE is widely recommended in clinical guidelines for risk stratification of advanced fibrosis in patients with MASLD (Eslam, Sarin, et al., 2020; Rinella et al., 2023; Tacke et al., 2024). The FIB-4 is a recommended initial assessment for advanced fibrosis in primary care due to its low cost, simplicity of calculation, and greater accuracy compared to most simple fibrosis scores [32]. However, FIB-4 has limitations in screening for advanced fibrosis, particularly among patients with T2DM, and it is also associated with a significant proportion of indeterminate results, necessitating additional tests such as VCTE to improve its accuracy [18,33]. VCTE has been widely accepted and validated in MASLD patients for diagnosing advanced fibrosis. The cutoff of LSM ≥ 10 kPa for advanced fibrosis has a corresponding sensitivity of 72.7% and specificity of 56.3% for F3–F4 fibrosis [34]. Currently, VCTE is not available in most primary care settings in Malaysia, and the practicality of this recommendation is debatable. In Malaysia, only seven VCTE machines are accessible in public healthcare settings (four public tertiary hospitals and three teaching hospitals) and none within primary care. Meanwhile, in private centres, patients are required to pay out of pocket, with fees ranging from MYR 300 to MYR 800 for the VCTE assessment [13,35]. With the recently updated Malaysian minimum monthly income set at MYR1,700, the cost of a single VCTE test could represent a substantial portion of a low-income individual’s monthly earnings, potentially posing a barrier to access [36]. A refinement to the stratification process, such as considering second-tier testing before the referral for VCTE assessment, should be explored to enhance stratification accuracy without incurring excessive additional costs.

Several studies in the UK, US, and Canada have reported cost analysis on the referral pathways among MASLD patients using first-tier FIB-4 testing, followed by second-tier testing in indeterminate cases with VCTE, enhanced liver fibrosis (ELF), magnetic resonance elastography (MRE) or shear wave elastography (SWE), highlighting that a two-step approach reduces referrals up to 70% and enhances cost savings range from 25% to 40% [37–40]. A Danish study reported a post hoc cost analysis of screening pathways using FIB-4, ELF, and VCTE. It showed that adding ELF as a second-tier test for indeterminate FIB-4 cases before the VCTE test helped save nearly 50% of costs and reduced over-referrals by more than 70%. [41]. This finding is consistent with our observation that the sequential FIB-4/GGT pathway could save more than 40% of the total cost and reduce over-referrals by 77% when the GGT serum test was added to indeterminate FIB-4 cases.

The majority of studies were conducted in countries with available resources, while the accessibility and affordability of these tests in resource-limited settings have not been studied. This is particularly concerning for countries with a high prevalence of MASLD, such as those in South America, Asia, North Africa, and the Middle East, many of which are still developing [5]. A cost-effectiveness analysis of MASLD screening in T2DM conducted by neighbouring country Singapore highlighted a similar issue regarding potential resource constraints and lack of access to VCTE in primary care, despite FIB-4 and VCTE being cost-effective strategies in their analysis [42]. Therefore, the selection of the test for the sequential pathway should rely on local availability to ensure feasibility within clinical practice.

Based on our previous study of the FIB-4 indeterminate group among T2DM patients with MASLD, GGT levels above the upper limit of normal (>ULN) were associated a 8.14-fold increased risk of advanced fibrosis (OR 8.14, 95% CI 2.41–27.52, p < 0.001) [15]. Our findings align with two local studies, which demonstrate that elevated GGT levels are associated with the presence of advanced liver fibrosis [13,20]. Lee et al. reported that T2DM patients with elevated GGT levels were 8.39 times more likely to develop advanced fibrosis (95% CI: 4.20–16.78). Similarly, Zain et al. reported that serum GGT > ULN was associated with a 9-fold increased risk (OR 9.38, 95% CI: 1.30–67.65; P = 0.026). The proposed solution to add a second-tier blood-based GGT test in indeterminate FIB-4 cases prior to referral to tertiary care, which is relatively inexpensive and more accessible in primary care, should be strongly considered.

We acknowledged that the sequential FIB-4/GGT pathway may result in a slightly higher rate of missed advanced fibrosis cases (15.5%) compared to 12.9% in the CPG pathway. We expect that these patients will be identified at a later date through routine follow-up using the same non-invasive testing pathway. Given that fibrosis progression occurs over a median of 14.3 years in steatosis and 7.1 years in steatohepatitis, routine monitoring could provide a safety net that enables the subsequent detection of disease progression. [43]. Emerging evidence suggests the value of longitudinal FIB-4 monitoring, whereby increases over time are associated with fibrosis progression, worsening liver stiffness, and a higher risk of adverse clinical outcomes, including mortality and liver-related events [44]. While FIB-4 is not intended as a standalone tool for risk prediction, substantial changes in FIB-4 values greater than 1.5 may help identify disease progression, with a reported positive predictive value of 59% and a negative predictive value of 72% [32,45,46]

This study has several strengths. To the best of our knowledge, it is the first study to analyse the cost-effectiveness of advanced fibrosis referral pathways in Malaysia. This contributes to the existing body of knowledge, as cost analysis data on advanced fibrosis detection pathways, particularly in resource-limited countries, are scarce. Secondly, the analysis focused on the T2DM population with MASLD, rather than the general population, which aligns with the current recommendation to screen high-risk populations for the early detection of advanced fibrosis, a stage significantly associated with high risk of liver-related mortality [33]. Thirdly, the decision-analytical model was validated with input from Ministry of Health experts across various disciplines, including hepatology/gastroenterology, endocrinology, and family medicine, to ensure it accurately represents the public healthcare landscape in Malaysia. This enhances the relevance and applicability of the findings for local policy and clinical practice. While our findings may be geographically specific to Malaysia, the results offer valuable insights for policymakers in other countries with similarly resource-constrained healthcare systems to consider their referral pathways. Lastly, we presented a CEAC that helps frame the uncertainty in decision-making and provides information on the probability of cost-effectiveness for each pathway across a range of willingness-to-pay threshold values [47]. As there is no consensus on the WTP threshold for detecting advanced fibrosis, the acceptability threshold for balancing performance accuracy with cost considerations enables policymakers to make informed choices based on the presented evidence.

The model input for our analysis was obtained from three different studies, with no single source covering the entire testing sequence and outcomes, which may influence the decision outcome due to heterogeneity. We addressed this limitation by prioritising data from populations where the same sequence of tests was performed, accounting for conditional dependence between tests (e.g., ALT followed by VCTE, FIB-4 followed by VCTE, and FIB-4 followed by GGT and then VCTE). Additional published literature on NITs for identifying advanced fibrosis within similar populations was reviewed with experts to ensure the inclusion of the best probability estimates. Secondly, variations in FIB-4 threshold cutoffs for risk categories existed across studies; low-risk thresholds ranged from <1.3 to <1.45, indeterminate risk from 1.3 to 2.67 or 1.3 to 3.25, and high-risk from >2.67 or >3.25. We assumed that these slight differences in cutoffs are unlikely to substantially influence model outcomes; if they do, all uncertainties are evaluated through sensitivity analyses. Thirdly, our model did not include newer non-invasive tests such as MAF-5, LiverPRO, LiverRisk, and SAFE score, as some are not yet widely validated across diverse populations, are not routinely implemented in clinical workflows, or involve parameters that may limit immediate uptake in resource-limited primary care settings [48]. Similarly, commercial serum tests, such as ELF, PRO-C3-based score (ADAPT), Hepascore, and FibroMeter were not included due to their high cost and unavailability in local settings. Lastly, our analysis focused solely on the short-term cost-effectiveness of the referral pathways, similar to previous studies that measured effectiveness by correct diagnoses [39,40]. We used “advanced fibrosis cases identified” over “correct diagnoses” since performance differences were mainly driven by the number of true negatives (non-referred cases- no advanced fibrosis).

In conclusion, the sequential FIB-4/GGT pathway is potentially the most cost-effective approach for identifying advanced fibrosis among T2DM patients with MASLD in our primary care setting. This sequential approach could potentially reduce tertiary care referrals and lower healthcare resource use and costs, while maintaining comparable effectiveness to other existing pathways. It is a pragmatic solution for resource-constrained settings, where financial constraints often restrict what can be realistically implemented in real-world practice. The CPG pathway was potentially more effective than current practice, but incurred higher costs and required substantially higher availability of VCTE compared to what is presently available within clinical practice.

## Supporting information

S1 Appendix CHEERS checklist. (DOCX)

S1 Table Input parameters used in the decision analytical model. (PDF)

S2 Table One-way deterministic sensitivity analysis based on ICER of cost/advanced fibrosis cases identified (CPG Pathway vs. Current Practice). (PDF)

S3 Table One-way deterministic sensitivity analysis based on ICER of cost/advanced fibrosis cases identified (Sequential FIB-4/GGT Pathway vs. Current Practice). (PDF)

S1 Fig Sensitivity analysis of ICER across a range of proportions of advanced fibrosis given ALT ≥ ULN- CPG Pathway vs. Current Practice. ALT, alanine transaminase; CPG, clinical practice guidelines; ICER, incremental cost-effectiveness ratio; LSM, liver stiffness measurement; ULN, upper limit of normal.
(TIF)

S2 Fig Sensitivity analysis of ICER across a range of proportions of FIB-4 ≥ 3.25- Sequential FIB-4/GGT Pathway v.s. **Current Practice.** FIB-4, Fibrosis-4; GGT, gamma-glutamyl transferase; ICER, incremental cost-effectiveness ratio.
(TIF)
