# Supplementary material for: Cost-effectiveness of various referral pathways to identify advanced fibrosis among type 2 diabetes mellitus patients with metabolic dysfunction-associated steatotic liver disease in primary care setting in Malaysia
Source: PLoS One. 2026 May 28;21(5):e0350263. doi: 10.1371/journal.pone.0350263 (PMC13218488; doi:10.1371/journal.pone.0350263)
Supplement: S1 Table — (PDF) [file pone.0350263.s002.pdf]

S1 Table. Input parameters used in the decision analytical model

Parameters	Base Case	Min	Max	Source/ Remarks
**Event Characteristics**				
Proportion of FIB-4<1.3	0.673	0.517	0.798	**Institute for Public Health, 2023 (Unpublished raw data)**<br>*Min and Max using a 95% confidence interval
Proportion of FIB-4 1.3-3.25	0.300	0.182	0.452	
Proportion of FIB-4≥3.25	0.027	0.007	0.101	
Proportion of ALT≥ULN	0.246	0.134	0.357	
Proportion of GGT≥ULN given FIB-4 1.3-3.25	0.507	0.278	0.810	
Proportion of LSM≥10kPa given FIB-4≥1.3	0.430	0.323	0.538	**Chan WK et al, 2019 (under supplementary data)**<br>*Min and Max using a 25% variance from the base value
Proportion of LSM<10kPa given FIB-4<1.3	0.809	0.607	1.000	
Proportion of LSM≥10kPa given FIB-4≥3.25	0.542	0.407	0.678	
Proportion of LSM≥10kPa given ALT≥ULN	0.510	0.383	0.638	**Lai et al, 2019 (Unpublished raw data)**<br>*Min and Max using a 25% variance from the base value
Proportion of LSM<10kPa given ALT<ULN	0.794	0.596	0.993	
Proportion of LSM≥10kPa given GGT≥ULN in FIB-4 1.3-3.25	0.800	0.600	1.000	
Proportion of LSM<10kPa given GGT<ULN in FIB-4 1.3-3.25	0.826	0.620	1.000	
**Resource Use**	**Costs, MYR in 2024**			**Source/ Remarks**
	**Base Case**	**Min**	**Max**	
**Outpatient Visit**				**Local Cost Data (Azzeri A, 2014)**<br>*Min and Max using a 25% variance from the base value
Tertiary care outpatient visit	147.11	110.33	183.89	
**Radiology**				
Vibration-controlled transient elastography (VCTE)	340.60	255.45	425.75	
**Lab Investigation**				
Full blood count (FBC)	10.72	8.04	13.40	
Liver function test (LFT)	7.30	5.48	9.13	
Aspartate aminotransferase (AST)	7.10	5.33	8.88	
Gamma-glutamyl transferase (GGT)	7.27	5.45	9.09	
**Stratification Strategy**	**Costs, MYR in 2024**			**Assumption**
	**Base Case**	**Min**	**Max**	
FIB-4 (new case at primary care)	25.12	18.84	31.40	Parameters needed for calculation: Age, platelet, AST, ALT
FIB-4 (routine care at primary care)	7.10	5.33	8.88	Assumed FBC and LFT are already available under routine care, only charge 1 additional test for AST
FIB-4 and VCTE (new case at tertiary care)	659.94	494.96	824.93	2 tertiary care visits + 1 VCTE + 1 blood test for FIB-4
Sequential FIB-4/GGT (both at primary care)	14.37	10.78	17.96	Blood tests for AST + GGT
GGT (at primary care)	7.27	5.45	9.09	One blood test for GGT
No stratification (referral based on ALT)	0	-	-	No additional cost

Cost in 2024 Malaysian Ringgit (MYR).

ALT, alanine aminotransferase; AST, Aspartate Aminotransferase; FBC, full blood count; FIB-4, fibrosis-4 index; GGT, gamma-glutamyl transferase; LFT, liver function test; LSM, liver stiffness measurement; ULN, upper limit of normal; VCTE, vibration-controlled transient elastography
