# Supplementary material for: Cost-effectiveness of various referral pathways to identify advanced fibrosis among type 2 diabetes mellitus patients with metabolic dysfunction-associated steatotic liver disease in primary care setting in Malaysia
Source: PLoS One. 2026 May 28;21(5):e0350263. doi: 10.1371/journal.pone.0350263 (PMC13218488; doi:10.1371/journal.pone.0350263)
Supplement: S2 Table — (PDF) [file pone.0350263.s003.pdf]

S2 Table. One-way deterministic sensitivity analysis based on ICER of cost/advanced fibrosis cases identified (CPG Pathway vs. Current Practice)

Parameters	Incremental Cost (min)	Incremental Cost (base)	Incremental Cost (max)	Incremental Effect (min)	Incremental Effect (base)	Incremental Effect (max)	ICER (min)	Interpretation of ICER (min)	ICER (base)	ICER (max)	Interpretation of ICER (max)	Absolute ICER (max-min)	Description
p_lsm≥10_given_ALT≥ULN (base 0.510, min 0.383, max 0.638)	60,555	60,555	60,555	47	16	-16	1,288	more effective, same cost	3,785	-3,785	less effective, same cost	5,073	Proportion of LSM≥10kPa given ALT≥ULN
p_lsm≥10_given_fib4≥1.3 (base 0.430, min 0.323, max 0.538)	60,555	60,555	60,555	-19	16	51	-3,187	less effective, same cost	3,785	1,187	more effective, same cost	4,374	Proportion of LSM ≥10kPa given FIB-4 ≥1.3
p_ALT≥ULN (base 0.246, min 0.134, max 0.357)	134,468	60,555	-12,698	73	16	-41	1,842	more effective, more costly	3,785	310	less effective, less costly	1,532	Proportion of ALT≥ULN
p_fib4<1.3 (base 0.673, min 0.517, max 0.798)	163,506	60,555	-21,937	83	16	-38	1,970	more effective, more costly	3,785	577	less effective, less costly	1,393	Proportion of FIB-4 <1.3
c_VCTE (base 341, min 255, max 426)	53,658	60,555	67,452	16	16	16	3,354	same effective, less costly	3,785	4,216	same effective, more costly	862	Cost of VCTE
c_TC_clinic_visit (base 147, min 110, max 184)	54,597	60,555	66,514	16	16	16	3,412	same effective, less costly	3,785	4,157	same effective, more costly	745	Cost of tertiary care clinic visit
c_FIB-4 (base 25, min 19, max 31)	60,046	60,555	61,064	16	16	16	3,753	same effective, less costly	3,785	3,816	same effective, more costly	64	Cost of FIB-4

All cost values are 2024 Malaysia Ringgit.

ALT, alanine transaminase; CPG, clinical practice guidelines; FIB-4, Fibrosis-4; GGT, gamma-glutamyl transferase; ICER, incremental cost-effectiveness ratio; LSM, liver stiffness measurement; TC, tertiary care; ULN, upper limit of normal; VCTE, vibration-controlled transient elastography
