# Supplementary material for: Cost-effectiveness of various referral pathways to identify advanced fibrosis among type 2 diabetes mellitus patients with metabolic dysfunction-associated steatotic liver disease in primary care setting in Malaysia
Source: PLoS One. 2026 May 28;21(5):e0350263. doi: 10.1371/journal.pone.0350263 (PMC13218488; doi:10.1371/journal.pone.0350263)
Supplement: S3 Table — (PDF) [file pone.0350263.s004.pdf]

S3 Table. One-way deterministic sensitivity analysis based on ICER of cost/advanced fibrosis cases identified (Sequential FIB-4/GGT Pathway vs. Current Practice).

Parameters	Incremental Cost (min)	Incremental Cost (base)	Incremental Cost (max)	Incremental Effect (min)	Incremental Effect (base)	Incremental Effect (max)	ICER (min)	Interpretation of ICER (min)	ICER (base)	ICER (max)	Interpretation of ICER (max)	Absolute ICER (max-min)	Description
p_fib4>3.25 (base 0.027, min 0.007, max 0.101)	-48,276	-34,935	14,426	1	12	52	-48,276	less effective, less costly	-2,911	277	more effective, more costly	48,553	Proportion of FIB-4 ≥3.25
p_lsm≥10_given_GGT≥ULN_in_fib4ind (base 0.800 , min 0.600, max 1.000)	-34,935	-34,935	-34,935	-19	12	42	1,839	less effective, same cost	-2,911	-832	more effective, same cost	2,670	Proportion of LSM ≥10kPa given GGT≥ULN in FIB-4≥1.3-<3.25
p_lsm≥10_given_ALT≥ULN (base 0.510, min 0.383, max 0.638)	-34,935	-34,935	-34,935	43	12	-20	-812	more effective, same cost	-2,911	1,747	less effective, same cost	2,559	Proportion of LSM≥10kPa given ALT≥ULN
p_lsm≥10_given_fib4≥3.25 (base 0.542, min 0.407, max 0.678)	-34,935	-34,935	-34,935	8	12	15	-4,367	less effective, same cost	-2,911	-2,329	more effective, same cost	2,038	Proportion of LSM ≥10kPa given FIB-4 ≥3.25
p_fib4ind (base 0.300, min 0.182, max 0.452)	-76,227	-34,935	18,065	-36	12	73	2,117	less effective, less costly	-2,911	247	more effective, more costly	1,870	Proportion of FIB-4≥1.3-<3.25
p_ALT≥ULN (base 0.246, min 0.134, max 0.357)	38,978	-34,935	-108,188	69	12	-45	565	more effective, more cost	-2,911	2,404	less effective, less costly	1,839	Proportion of ALT≥ULN
p_GGT>ULN_given_fib4ind (base 0.507, min 0.278, max 0.810	-80,471	-34,935	25,120	-44	12	84	1,829	less effective, less costly	-2,911	299	more effective, more costly	1,530	Proportion of GGT≥ULN given FIB-4≥1.3-<3.25
c_VCTE (base 341, min 255, max 426)	-29,230	-34,935	-40,640	12	12	12	-2,436	same effective, more costly	-2,911	-3,387	same effective, less costly	951	Cost of VCTE
c_TC_clinic_visit (base 147, min 110, max 184)	-30,006	-34,935	-39,864	12	12	12	-2,501	same effective, more costly	-2,911	-3,322	same effective, less costly	821	Cost of tertiary care clinic visit
p_fib4<1.3 (base 0.673, min 0.517, max 0.798)	-36,043	-34,935	-34,047	12	12	12	-3,004	same effective, less costly	-2,911	-2,837	same effective, more costly	166	Proportion of FIB-4 <1.3
c_GGT (base 7, min 5, max 9)	-35,481	-34,935	-34,389	12	12	12	-2,957	same effective, less costly	-2,911	-2,866	same effective, more costly	91	Cost of GGT
c_FIB-4 (base 25, min 19, max 31)	-34,514	-34,935	-35,356	12	12	12	-2,876	same effective, more costly	-2,911	-2,946	same effective, less costly	70	Cost of FIB-4

All cost values are 2024 Malaysia Ringgit. ALT, alanine transaminase; CPG, clinical practice guidelines; FIB-4, Fibrosis-4; GGT, gamma-glutamyl transferase; ICER, incremental cost-effectiveness ratio; LSM, liver stiffness measurement; TC, tertiary care; ULN, upper limit of normal; VCTE, vibration-controlled transient elastography
